# Disruption of Cigarette Smoking Addiction After Dorsal Striatum Damage

**DOI:** 10.3389/fnbeh.2021.646337

**Published:** 2021-04-01

**Authors:** Chuya Jing, Changxin Jing, Liangcheng Zheng, Ganji Hong, Jingjing Zheng, Lu Yu, Ningning Song, Tengkun Zhang, Qilin Ma, Jie Fang

**Affiliations:** ^1^Department of Neurology, The First Affiliated Hospital of Xiamen University, Xiamen, China; ^2^Department of Endocrinology, The First Hospital of Yulin, Yulin, China; ^3^Graduate School of Fujian Medical University, Fuzhou, China

**Keywords:** cigarette smoking, addiction, disruption, dorsal striatum, behavioral

## Abstract

Studies have shown that addictive behavior is associated with many brain regions, such as the insula, globus pallidus, amygdala, nucleus accumbens, and midbrain dopamine system, but only a few studies have explored the role of the dorsal striatum in addictive behavior. In June 2020, we started contacting 608 patients who were hospitalized between January 2017 and December 2019, and we recruited 11 smoking addicts with dorsal striatum damage and 20 controls with brain damage that did not involve the dorsal striatum (the damaged areas included the frontal lobe, temporal lobe, parietal lobe, brain stem, thalamus, internal capsule, and so on). All study participants had brain damage due to acute cerebral infarction. Disruption of smoking addiction was found to be significantly associated with the dorsal striatum (Phi = 0.794770, *P* = 0.000015). Our findings suggested that patients in the dorsal striatum group were more likely to discontinue smoking than those in the non-dorsal striatum group. The characteristics of this interruption is that smoking can be quit more easily and quickly without recurrence and that the impulse to smoke is reduced. These results suggest that the dorsal striatum is a key area for addiction to smoking.

## Introduction

Addiction is a pathological process that involves learning and memory. Potentially addictive drugs activate the brain's reward system, adaptively changing the structure and function of the nerves in this part of the brain. Research on addiction, which has increased in the past 10 years, indicates that many areas of the brain are involved in the addiction pathway, including the insula, globus pallidus, amygdala, nucleus accumbens, ventral striatum, frontostriatal, and the midbrain dopamine system (Volkow et al., 2006; Naqvi et al., 2007; Wang et al., 2007; Koob and Volkow, 2010; Hefzy et al., 2011; Yuan et al., 2018).

Recent evidence indicates that the dorsal striatum plays an important role in addiction. Several studies have shown that dorsal striatum activity increases in response to drug cues relative to neutral cues in drug users (Vollstädt-Klein et al., 2010; Claus et al., 2011; Schacht et al., 2011). A study by McClernon et al. had 18 adult smokers undergo fMRI scanning following two conditions: smoking as usual and a 24-h abstinence period. After abstinence, greater fMRI activity was observed in response to smoking cues compared to control cues in the dorsal striatum. The same effect was also observed in the parietal, frontal, occipital, and central cortical regions, and the thalami (McClernon et al., 2009). Vollstadt-Klein and colleagues also reported that dorsal striatum activity in response to drug cues was positively correlated with drug craving in heavy drinkers (Vollstädt-Klein et al., 2010). A study by Janes et al. reported that nicotine-dependent smokers who failed to quit smoking showed greater cue-induced activity in the dorsal striatum, among other regions, compared to smokers who remained abstinent (Janes et al., 2010). Zhou et al. found that heavy cannabis users selectively exhibited dorsal striatal reactivity (Zhou et al., 2019). Another study found that the volume of the putamen was positively correlated with the duration of abstinence in former regular users of alcohol who were abstinent for a long time (Korponay et al., 2017). A clinical trial showed that damage to only the dorsal striatum can cause disruption of smoking addiction, and when basal ganglia damage is combined with insula damage, the disruption increases (Gaznick et al., 2014). Furthermore, dorsal striatum connectivity with the cingulo-insular network was found to be associated with smoking cessation (Sweitzer et al., 2016).

Against this background, because smoking is one of the most common addictions in China, and there are few clinical studies that have investigated the relationship between the dorsal striatum and smoking addiction, our research has tried to understand the role of the dorsal striatum in the pathway of smoking addiction. As acute cerebral infarction is one of the most important causes of brain damage treated in a neurology department, we recruited patients who had an acute cerebral infarction, of whom, 11 only had damage to the dorsal striatum and 20 had brain damage that did not involve the dorsal striatum. Participants answered a series of questions about their smoking history, and their results were compared to investigate the relationship between the dorsal striatum and smoking addiction.

## Materials and Methods

All of the study participants were recruited between January 2017 and December 2019 from the First Affiliated Hospital of Xiamen University, Xiamen, China. All the procedures were reviewed and approved by the First Affiliated Hospital of Xiamen University, and all the subjects provided signed informed consent prior to participating in the study. We reviewed the patients' electronic records to ensure they met the following inclusion criteria: (1) they suffered an acute cerebral infarction; (2) their lesions could be visualized using MRI; (3) they had a smoking history; (4) they did not suffer from amnesia or severe aphasia; and (5) they were not addicted to drugs other than nicotine at the time of lesion onset, per their medical records.

### Subject Selection

**Figure 1:**
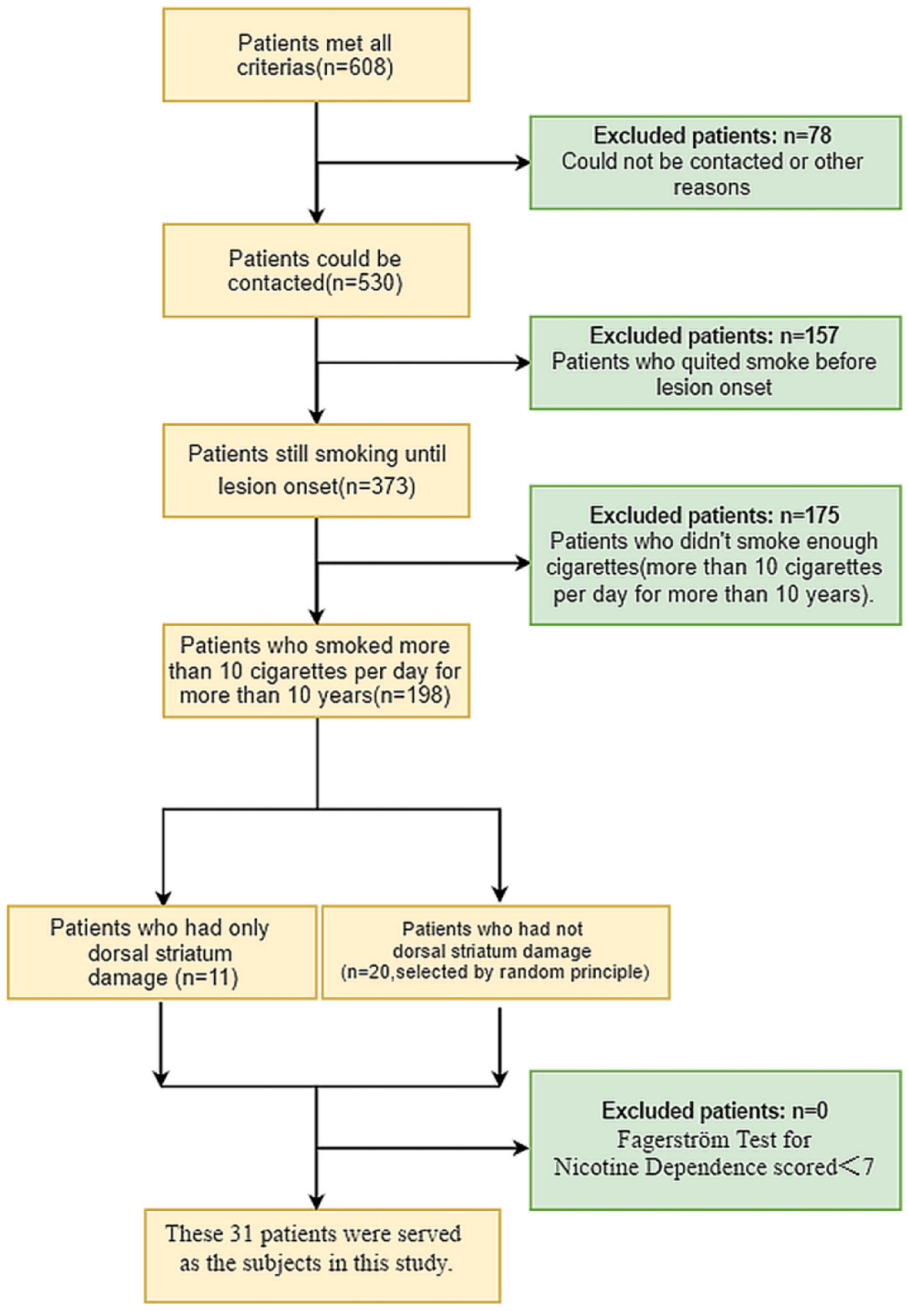
Flow chart of patient inclusion.

Starting in June 2020, we attempted to contact 608 patients who met these inclusion criteria to determine their smoking history: 78 patients could not be contacted (because their telephone numbers had changed, they died, or some other reason), and 157 patients reported they smoked at some time, but quit a number of years before lesion onset. Of the 373 remaining patients, 198 reported that they smoked more than 10 cigarettes per day for more than 10 years at the time of lesion onset. Among these 198 patients, 11 had only dorsal striatum damage and 20 had non-dorsal striatum damage (The damaged regions included the thalamus, internal capsule, caudate nucleus, and other regions in the brain, and some patients had damage in more than one region). Each of these 31 patients was asked to complete the Fagerström Test for Nicotine Dependence (FTND), which is the most valid and commonly used scale for measuring nicotine addiction (Fagerström, 1978). Scores on the test range from 0 to 10, with the higher scores indicating stronger smoking dependence. Nicotine addiction was categorized as low nicotine dependence (0–4 points), moderate nicotine dependence (5–6 points), and high nicotine dependence (7–10 points). All 31 of the patients scored 7–10 points, which means they were highly nicotine dependent. These 31 patients finally served as the subjects in this study, and were included in the statistical analysis. A flow chart of patient inclusion is shown in Figure 1.

### Measures

We obtained relevant information about the subjects through electronic records and interviews, including their sex, current age, age at lesion onset, years of smoking at lesion onset, and number of cigarettes smoked per day at lesion onset. Additional smoking measures are described in section Behavioral Classification.

### Statistical Analysis

Group differences in demographic characteristics and detailed smoking data were analyzed by SPSS Version 20.0 (IBM SPSS Statistics, Armonk, NY). Specifically, the means of the dorsal striatum group and non-dorsal striatum group were compared at baseline on the following variables: sex composition (i.e., number of males and females) current age, age at lesion onset, years of smoking at lesion onset, and number of cigarettes smoked per day at lesion onset. An independent two-sample *t*-test was used to analyze continuous variables, and Fisher's exact probability test was used for proportions. *P* < 0.05 was considered to be statistically significant. The effect sizes are reported as Phi coefficients (0.10 indicates a small effect, 0.3 indicates a medium effect, and 0.5 indicates a large effect).

### Behavioral Classification

The 31 patients were interviewed in order to determine how their smoking behavior changed after lesion onset (Information was obtained from relatives when necessary). All the patients were asked whether or not they had smoked in the past 6 months. Patients who reported they smoked during the past 6 months were classified as “non-quitters.” Those who reported they did not smoke during the past 6 months were classified as “quitters.” According to the classification method of Naqvi et al. (2007), all of the “quitters” were asked some further questions in order to understand their experience of quitting smoking in relation to the onset of their lesions. The questions were as follows: (1) “How soon after your brain injury did you quit smoking?”; (2) “How difficult was it to quit smoking after your brain injury (on a scale of 1–7, with one being very easy and seven being very difficult, the score is based on the subjective feelings of the patient)?”; (3) “How many times have you started smoking again since your brain injury?”; and (4) “Have you experienced any impulse to smoke again since you quit smoking?” Patients who reported they quit smoking <1 day after their brain injury, who rated their difficulty of quitting as <3, who reported they did not start smoking again since their brain injury, and reported that they felt no impulse to smoke again since quitting were classified as having a “disruption of smoking addiction.” The remaining patients were classified as having “no disruption of smoking addiction.”

### MRI Acquisition

A 3.0 T MRI system (Ingenia, Philips Medical Systems, Netherlands) was used for all data collection. The head coil had a 16-channel phased-array. Other imaging parameters were as follows: T1-weighted images (TR = 250 ms; TE = 2.3 ms; slices = 21; thickness = 6 mm; gap = 1 mm; FA = 75°; matrix = 256 × 163; FOV = 230 × 180 mm. NSA = 2. The sequence took 1 min and 23 s); T2-weighted images (TR = 2866 ms; TE = 120 ms; slices = 21; thickness = 6 mm; gap = 1 mm; FA = 90°;matrix = 358 × 299; FOV = 230 mm × 200 mm. NSA = 1.5. The sequence took 1 min and 37 s); Fluid attenuated inversion recovery (FLAIR) sequence (TR = 10000 ms; TE = 125 ms; slices = 21; thickness = 6 mm; gap = 1 mm; FA = 75°; acquisition matrix = 308 × 200; FOV = 230 × 200 mm. NSA = 1. Inversion recovery delay time = 2450 ms. The sequence took 2 min); Diffusion weighted imaging sequence with b-values = 1000 s/mm^2^ (TR = 4234 ms; TE = 78 ms; slices = 21; thickness = 6 mm; gap = 1 mm; FA = 90°; FOV = 230 × 230 mm; matrix = 152 × 122; voxel size = 1.5 × 1.9 × 6.0 mm^3^. NSA = 1. The sequence took 1 min and 54 s).

## Results

**Figure 2:**
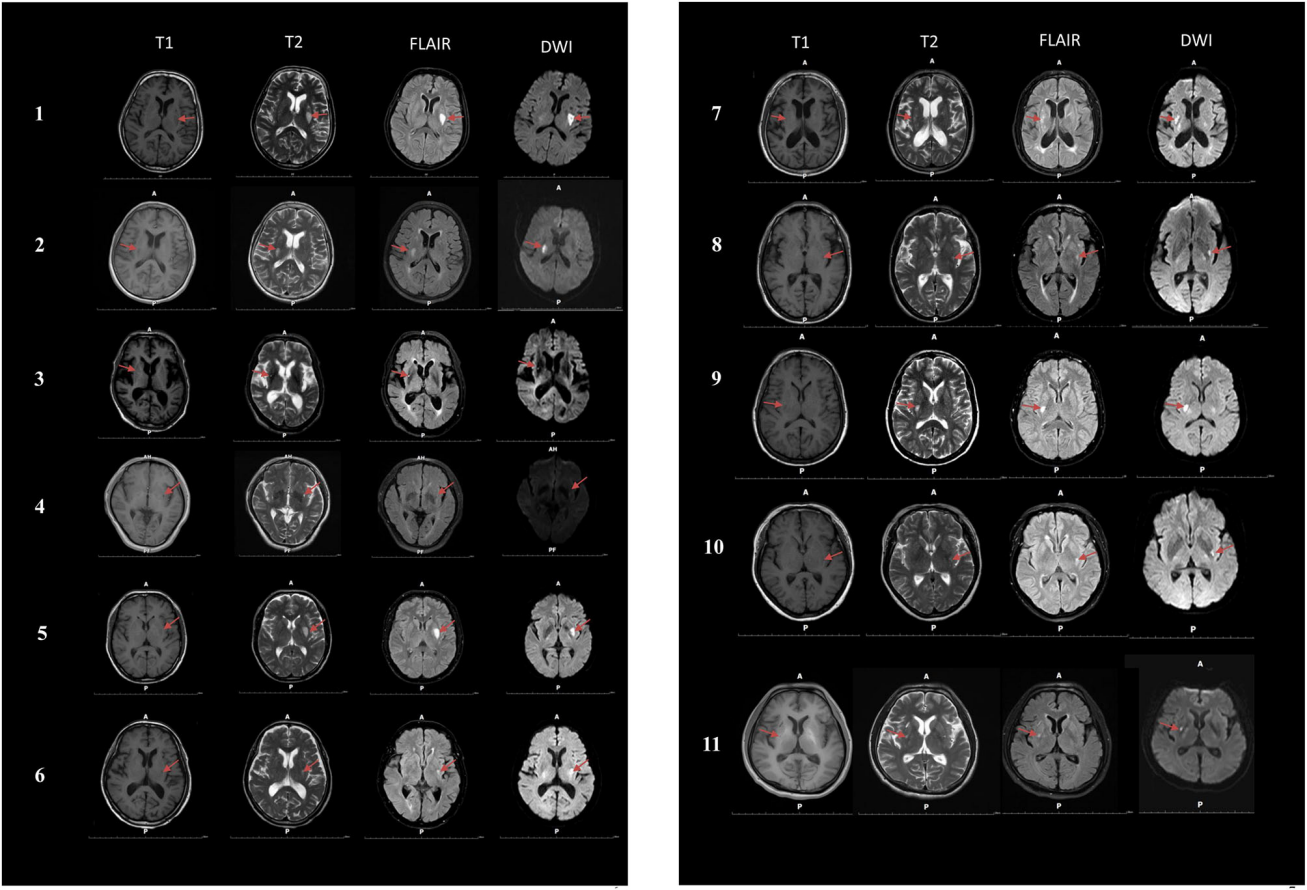
Magnetic resonance imaging (MRI) was performed on 11 smokers with dorsal striatum damage. T1, T1-weighted images; T2, T2-weighted images; FLAIR, Fluid attenuated inversion recovery; DWI, Diffusion weighted imaging.

Among the 11 cigarette smokers who had suffered only dorsal striatum damage, five had right dorsal striatum damage and six patients had left dorsal striatum damage (Figure 2). The means of the dorsal striatum group and non-dorsal striatum group did not differ significantly for sex composition, current age, age at lesion onset, years of smoking at lesion onset, or number of cigarettes smoked per day at lesion onset (*p* > 0.05) (Tables 1, 2).

**Table 1:** Detailed information about patients who acquired dorsal striatum damage.

**Number**	**Region**	**Gender**	**Age**	**Age at lesion onset**	**Years smoking at lesion onset**	**Cigarettes/day at lesion onset**
1	Left	Male	71	68	25	20
2	Right	Female	62	61	19	15
3	Right	Male	71	70	22	22
4	Left	Male	63	60	35	30
5	Left	Male	55	53	24	18
6	Left	Male	77	75	35	30
7	Right	Male	47	44	10	12
8	Left	Male	79	78	33	15
9	Right	Female	59	58	12	10
10	Left	Female	56	55	14	10
11	Right	Male	54	51	17	20

*Left, left dorsal striatum; Right, right dorsal striatum*.

**Table 2:** Characteristics of the dorsal striatum group and the non-dorsal striatum group.

	**Dorsal striatum (*n* = 11)**	**Non-dorsal striatum (*n* = 20)**	***t***	***P*-value**
Females (number)	4.00 (36.36%)	9.00 (45.00%)	–	0.718
Age (years)	63.09 ± 10.23	64.10 ± 10.58	−0.257	0.80
Age at lesion onset (years)	61.18 ± 10.55	61.85 ± 10.05	−0.174	0.86
Years smoking at lesion onset	22.36 ± 9.01	23.50 ± 8.90	−0.488	0.63
Cigarettes smoked per day at lesion onset	18.36 ± 7.08	18.70 ± 7.76	−0.119	0.91

**Figure 3:**
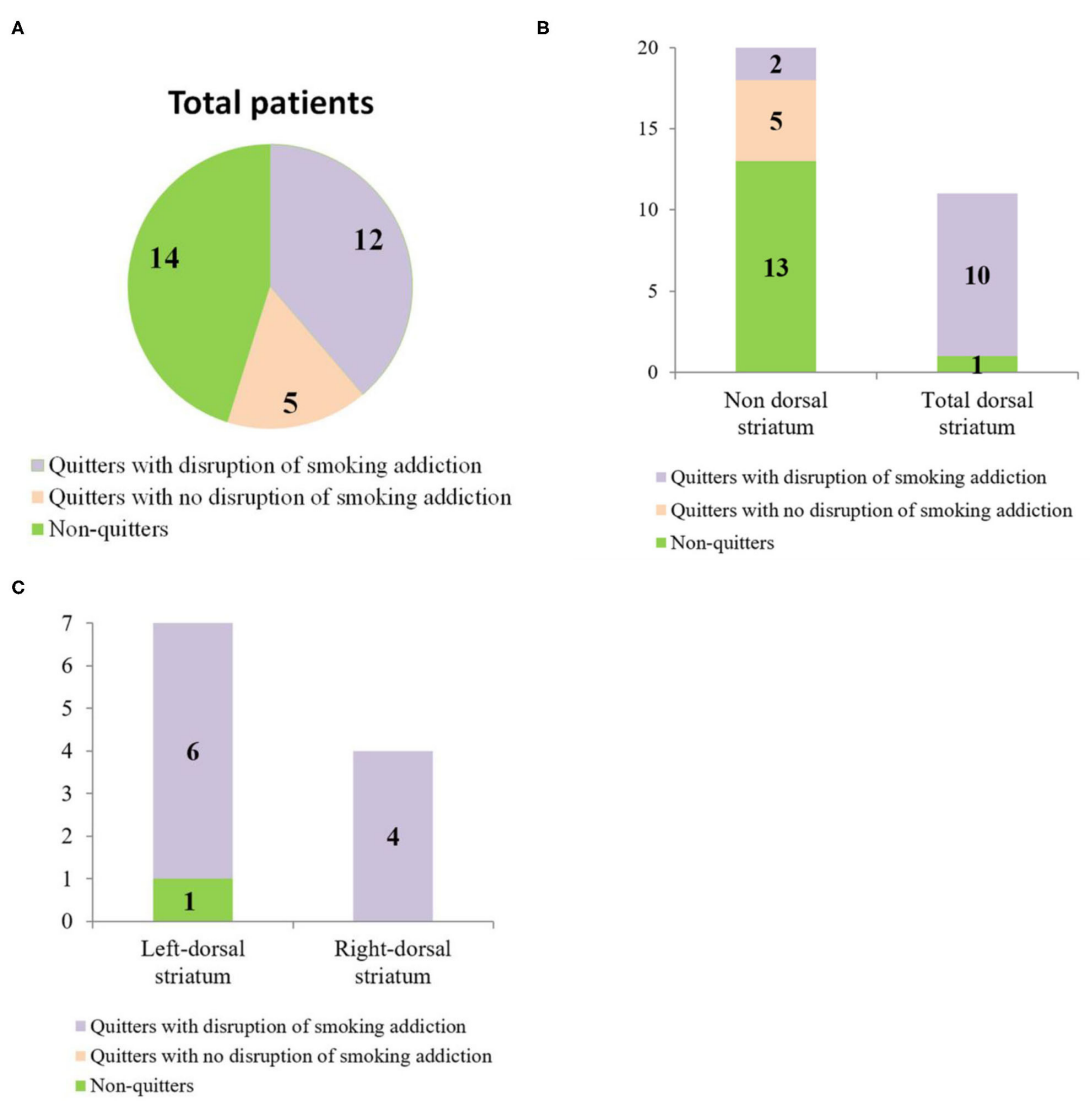
**(A)** Pie chart showing the behavioral classification of total patients. **(B)** Bar graph showing the number of patients in each anatomical group who fell into each of the behavioral categories. **(C)** Bar graph showing the number of patients with left dorsal striatum damage and right dorsal striatum damage who fell into each of the behavioral categories.

Based on the criteria described in section Behavioral Classification, 14 of the 31 patients were “non-quitters,” and 12 of the patients were “quitters” who quit smoking after lesion onset and met all four criteria for “having a disruption of smoking addiction.” The five remaining patients were “quitters” who failed to meet all four of these criteria, so they were classified as having “no disruption of smoking addiction” (Figure 3A). More details are provided in Figures 3B,C. In Table 3, the percentage of quitters with disruption of smoking addiction in the dorsal striatum group was 83.3%, which was much higher than 16.7% in the non-dorsal striatum group. Our findings showed that the likelihood of having a disruption of smoking addiction after a lesion in either the right or the left dorsal striatum was significantly higher than the likelihood of having a disruption of smoking addiction after a non-dorsal striatum lesion (Phi = 0.794770, *P* = 0.000015). When we examined the right and left dorsal striatum separately, we found that the likelihood of having a disruption of smoking addiction was significantly higher after a right dorsal striatum lesion than it was after a non-dorsal striatum lesion (Phi = 0.774597, *P* = 0.001412), and it was also significantly higher after a left dorsal striatum lesion than it was after a non-dorsal striatum lesion (Phi = 0.726641, *P* = 0.000608). There were two patients who had a disruption of smoking addiction after suffering brain damage that did not involve the dorsal striatum. When examined their lesions, each of them had damage in a unique set of regions. This raises the possibility that certain patients may have a disruption of smoking addiction as a general effect of suffering brain injury.

**Table 3:** Number of participants in different groups.

		**NQ/n(%)**	**NDSA/n(%)**	**DSA/n(%)**	**Phi**	***P*-value**
Dorsal striatum	left	1 (7.1%)	0 (0.0%)	6 (50.0%)	0.726641	0.000608
	right	0 (0.0%)	0 (0.0%)	4 (33.3%)	0.774597	0.001412
	total	1 (7.1%)	0 (0.0%)	10 (83.3%)	0.794770	0.000015
Non-dorsal striatum	total	13 (92.9%)	5 (100.0%)	2 (16.7%)	–	–

*NQ, Non-quitters; NDSA, Quitters with no disruption of smoking addiction; DSA, Quitters with disruption of smoking addiction*.

## Discussion

A review of the literature shows that few studies have examined the disruption of addiction after brain injury and most of those that have are case reports (Table 4). A study by Naqvi et al. of 69 patients which is one of the few studies to find a direct relationship between insula damage and disruption of smoking addiction. The results suggest that the insula is a critical neural substrate in the addiction to smoking (Naqvi et al., 2007). Recent evidence indicates that damage to the basal ganglia alone can cause disruption of smoking addiction, and when basal ganglia damage is combined with insula damage, the disruption increases (Gaznick et al., 2014).

**Table 4:** Some studies on the disruption of addiction after injury to different brain regions.

**Author**	**Participants**	**Etiologies**	**Lesion region**	**Addictive substances**	**Addiction time**	**Outcome**
Miller et al. (2006)	1	Methadone overdose	Globus pallidus	Alcohol, LSD, marijuana,cocaine, opiates, and Ecstasy	>10 years	No longer experienced pleasure from drugs and four serial urine toxicology screens were negative for 6 months.
Béchir et al. (2010)	1	Acute hemorrhagic stroke	Posterior cingulate	Cigarettes	18 years	Continued to be abstinent after 12 months.
Muskens et al. (2012)	1	Ischemic stroke	Dorsal striatum	Cigarettes	>20 years	Continued to be abstinent after 3 months.
Moussawi et al. (2016)	1	Methadone overdose	Globus pallidus	Alcohol and opiates	Not mentioned	Continued to be abstinent after 10 years.
Gaznick et al. (2014)	63	Acute stroke	Basal ganglia and insula	Cigarettes	Not mentioned	Had significantly higher and more sustained rates of smoking cessation
Naqvi et al. (2007)	69	–	Insula	Cigarettes	>2 years	Likely to quit smoking easily.
Abdolahi et al. (2015)	156	Ischemic stroke	Insula	Cigarettes	Smoked at least one cigarette per day during the month prior to their stroke and at least 100 in their lifetime	Had a lower WSWS score and MNWS score, appeared to be less likely to use NRT during admission.

The ventral striatum has classically been considered to play an important role in addiction. Many studies have implicated the ventral striatum in the anticipation and immediate response to rewards (Hariri et al., 2006; Luijten et al., 2017), and that it is associated with abstinence-induced craving (David et al., 2005; Franklin et al., 2007; Wang et al., 2007). One study also found that 4h of abstinence can significantly increase craving and reduce regional cerebral blood flow in the ventral striatum (Franklin et al., 2018). Our findings indicate the dorsal striatum may also be involved in the addiction pathway. Our results indicate that smokers who acquired dorsal striatum damage were very likely to quit smoking easily and immediately and to remain abstinent. Moreover, smokers with dorsal striatum damage were far less likely to experience conscious urges to smoke after quitting. Based on the research literature, the dorsal striatum may be involved in addiction through the following pathways.

First, many studies have demonstrated dopamine release in response to cigarette smoking (Brody et al., 2006; Scott et al., 2007) and that nicotine intake is an important factor in dopamine release from smoking (Cumming et al., 2003; Marenco et al., 2004; Brody et al., 2006; Scott et al., 2007; Takahashi et al., 2008). Belin et al. found that the interactions between the ventral striatum and the dorsal striatum mediated by dopaminergic transmission play an important role in drug addiction (Belin and Everitt, 2008). In addition, Volkow et al.'s human neuroimaging studies also observed cocaine cue-induced increases in dopamine release in the dorsal striatum (Volkow et al., 2006). Cocaine cues have also been shown to elicit dorsal striatal dopamine release in animal studies (Ito et al., 2002). Therefore, we speculate that after dorsal striatum injury, dopamine release from the dorsal striatum induced by smoking cues is decreased, thus, blocking this addiction mechanism.

Second, the dorsal striatum is extensively linked to the orbitofrontal cortex (Fornito et al., 2013), and studies have confirmed that the orbitofrontal cortex is closely related to drug abuse and drug addiction (Kasanetz et al., 2013). The striatum directly receives the projection of glutamate neurons from the orbitofrontal cortex, and addictive behavior may enhance the projection of the glutamate system from the orbitofrontal cortex to the dorsal striatum through repeated stimulation. Glutamate activates the neurons in the dorsal striatum, thus, increasing the release of dopamine in the striatum, and the increase of dopamine in the striatum activates GABA projection neurons, which express D1 receptors (Surmeier et al., 2007). Therefore, the inhibitory GABA neurons projecting to the substantia nigra pars reticulata (SNr) increase, which can inhibit the function of the SNr. Then, the inhibitory GABA neurons projecting from the SNr to the thalamus are reduced. Furthermore, the release of glutamate from the thalamus to the cortex is increased, and the high level of glutamate in the cortex is projected to the dorsal striatum through the cortex-striatum pathway; thus, forming a positive feedback mechanism. Damage to the dorsal striatum may hinder this feedback mechanism, thereby blocking the addiction process.

Moreover, numerous studies have found that cortical and subcortical structures play an important role in automatic behavior and motor planning (Johnson-Frey, 2004; Johnson-Frey et al., 2005; Lewis, 2006). Subjects with damage in these brain regions usually exhibit different types of apraxia or deficits in general action planning and execution (Lewis, 2006). Dorsal striatum circuits are known to interact with thalamic-cortical circuits that are involved in the planning and execution of motor responses. Smoking behavior becomes highly automatic after repeated practice in people with smoking addiction. We speculate that damage to the dorsal striatum may block the connection between the dorsal striatum and the thalamus, thus, impairing the planning and execution of smoking.

Our findings suggest that the dorsal striatum may play an important role in the process of smoking addiction. Therapies that modulate the function of the dorsal striatum may be useful to help smokers quit. However, we acknowledge that our sample sizes were too small. Thus, we look forward to more clinical studies in the future to provide more detailed explanations of the processes involved in addiction.

## Data Availability Statement

The raw data supporting the conclusions of this article will be made available by the authors, without undue reservation.

## Ethics Statement

The studies involving human participants were reviewed and approved by The Ethics Committee of The First Affiliated Hospital of Xiamen University. The patients/participants provided their written informed consent to participate in this study. Written informed consent was obtained from the individual(s) for the publication of any potentially identifiable images or data included in this article.

## Author Contributions

ChuJ performed the statistical analysis. ChangJ drafted the manuscript. LZ, GH, JZ, LY, NS, and TZ carried out the acquisition of data. QM helped to draft the manuscript. JF conceived the study, and participated in its design and coordination. All authors read and approved the final manuscript.

## Conflict of Interest

The authors declare that the research was conducted in the absence of any commercial or financial relationships that could be construed as a potential conflict of interest.
